# Agrobot Lala—An Autonomous Robotic System for Real-Time, In-Field Soil Sampling, and Analysis of Nitrates

**DOI:** 10.3390/s22114207

**Published:** 2022-05-31

**Authors:** Goran Kitić, Damir Krklješ, Marko Panić, Csaba Petes, Slobodan Birgermajer, Vladimir Crnojević

**Affiliations:** BioSense Institute—Research Institute for Information Technologies in Biosystems, University of Novi Sad, Dr. Zorana Đinđića 1a, 21000 Novi Sad, Serbia; dkrkljes@biosense.rs (D.K.); panic@biosense.rs (M.P.); chaba@biosense.rs (C.P.); b.sloba@biosense.rs (S.B.); crnojevic@biosense.rs (V.C.)

**Keywords:** UGV, precision agriculture, artificial intelligence, soil nutrient analysis, soil sampling

## Abstract

This paper presents an autonomous robotic system, an unmanned ground vehicle (UGV), for in-field soil sampling and analysis of nitrates. Compared to standard methods of soil analysis it has several advantages: each sample is individually analyzed compared to average sample analysis in standard methods; each sample is georeferenced, providing a map for precision base fertilizing; the process is fully autonomous; samples are analyzed in real-time, approximately 30 min per sample; and lightweight for less soil compaction. The robotic system has several modules: commercial robotic platform, anchoring module, sampling module, sample preparation module, sample analysis module, and communication module. The system is augmented with an in-house developed cloud-based platform. This platform uses satellite images, and an artificial intelligence (AI) proprietary algorithm to divide the target field into representative zones for sampling, thus, reducing and optimizing the number and locations of the samples. Based on this, a task is created for the robot to automatically sample at those locations. The user is provided with an in-house developed smartphone app enabling overview and monitoring of the task, changing the positions, removing and adding of the sampling points. The results of the measurements are uploaded to the cloud for further analysis and the creation of prescription maps for variable rate base fertilization.

## 1. Introduction

With the continuous growth of the world population, the demand for food and cultivated land increases continuously. The prediction of the Food and Agriculture Organization of United Nations (FAO) indicates the presence of constant growth of the population with the rate of 79 million people per year, increasing food demand [1]. Since the cultivated land resources are limited, and acquiring new ones is correlated with degradation of ecosystems, reduction of forests, climate changes, and risks of new pandemic breakouts, as well as degradation of soil properties due to inappropriate cultivation and treatment, there is an urgent need to improve soil treatment, to increase yield in a sustainable manner. The best approach that will enable farming to become more efficient in a sustainable way and reduce the production costs at the same time, is to provide an efficient supply of nutrients and water [2]. Standard and classical methods of soil analysis usually involve taking 1–20 samples per 5 hectares from around 30 cm depth. They are usually mixed and analyzed for an average value of nutrients [3]. A laboratory analysis then takes 10–15 days to obtain the results. The most common soil sampling methods used are hand sampling, hydraulic probes, electric probes, and auger probes [4]. Hand sampling is easy to use and economic, but it is time-consuming, labor-intensive, and could be inconsistent with the sampling depths. Hydraulic probes are fast and have a consistent depth, but are composed of numerous components (engine, hydraulics tank, pump, and lines), vendor locked, and pricy in the range from USD 4000 to 8000 on average. Electric probes demand low maintenance, with no fuel costs, and are more suited for dusty conditions. They do have a slower cycle time and they are not as powerful as hydraulic ones. Auger probes are the most durable and easy to set up and use. Their main drawback is the cross-contamination due to poor probe cleanout capabilities and they have difficulties with verifying core depth as well with their usage in sandy soils. All the stated sampling methods end up with time-consuming laboratory analysis, which does not provide on-time information to the farmers. Furthermore, due to sample averaging, precise information about nutrients at the exact location is lost. Considering nitrogen, with its large spatial and temporal variations, under and over-fertilization is inevitable. This can lead to a decrease in yield on one hand, and significant pollution of the ecosystems on the other [5]. 

Sowing and fertilization as phases in the agricultural production process should be completed after soil analysis based on appropriate methods providing information on nutrient availability for the growth and development of the particular plant. Until recently, farmers applied uniform fertilization per plot, which is not optimal from an economic perspective, and unsustainable from an ecologic, environmental, and ecosystem point of view. Plants acquire nutrients in amounts that are needed for normal growth, while any additional artificially applied nutrients by fertilization largely evaporate, creating greenhouse gases, or end up in surface and underground waters, which boosts algae growth in rivers and lakes [6]. Subsequently, lack of oxygen leads to large-scale fish death and other water animals and organisms in their natural habitats, which finally affects humans. All the stated facts impose a need for new systems and methods for real-time georeferenced sampling of soil for nutrients, providing input for precise fertilization.

Precision agriculture, smart farming, and automated agricultural technology have emerged as promising methodologies for increasing crop productivity without sacrificing produced quality. The emergence of various robotics technologies has facilitated the application of these techniques in agricultural processes [7]. In the stated article, the authors provide an overview of the current state of the art in agricultural robots. Classification of the five major operations in open arable farming is presented in the study by [8], where one of them is soil analysis. An implication of soil agrochemical analysis and its accuracy in precision agriculture is given in [9]. The authors suggest that rapid, less labor-intensive, economical, and at least equally accurate methods have to be developed for precision agriculture. They emphasize high soil analysis costs with traditional methods. A suggestion for scales of 10 m or less in precise agriculture is suggested in [8], due to the high spatial variability of the nutrients in the soil. Early work in the automation of soil sampling is presented in [10]. The system collects, packs, and marks samples with georeferencing. Rogo Robotic Soil Sampling [11] represents the state-of-the-art automatic soil sampler, however, it is not on-the-go, as it just collects and packs samples. The paper by Oledo et al. presented a UGV with a custom-built robotic manipulator with a surface scoop-type soil sampler [12]. Cao et al. presented a soil sampling system on a mobile robot with practical implementation in a soil survey in an assault zone [13]. The sampling system is an automatic penetrometer.

Vaeljaots et al. presented soil sampling UGV which can measure some of the soil properties online such as temperature, moisture, and penetration force [14]. It can collect, pack, and label the samples for later laboratory analysis. However, this paper does not present more technical insight, e.g., what are the exact parameters that are measured online, what is the depth of a sample, etc.

The robotic solutions mentioned above are only limited to the soil sampling part of the process and do not offer an insight into the nutrient analysis. In this paper, we propose a novel system for soil sampling and in-field analysis for nitrate-nitrogen that is based on the autonomous robotic platform agrobot Lala. The proposed solution is supported by an AI algorithm for defining optimal sampling locations as well as for fertilization prescription. In this way, a complete solution is offered to the farmers based on what they know on time, what is the spatial need for nitrogen in their fields, and how exactly to apply fertilizer. We believe that the proposed solution will help farmers be more efficient in a sustainable way.

## 2. Materials and Methods

**Figure 1:**
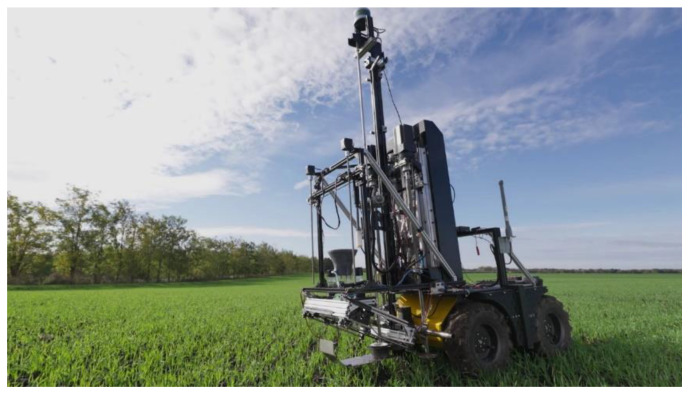
An autonomous robotic system—agrobot Lala.

The purpose of an autonomous robotic system, agrobot Lala (Figure 1) is an automation of the in-field, real-time, soil sampling and analysis of nutrient content of the soil. Currently, it supports the measurement of the content of nitrate nitrogen; however, with different probes it could measure other nutrients and soil properties as well (electric conductivity (EC), pH, NPK, and similar). Agrobot Lala is fully autonomous, and it is based on the commercially available UGV platform Husky (Clearpath robotics Inc., Kitchener, ON, Canada) [15]. This platform is equipped with a custom-made system for soil sampling and analysis. 

The system can be divided into three major parts:

- Cloud-based application for task management and generation of sample points on the plot based on a proprietary AI algorithm.
- Smartphone application for monitoring and customization of the task.
- Robotic system for acquiring and analysis of soil samples.

### 2.1. Cloud-Based Application

The cloud-based application is a custom-made proprietary solution based on the existing AgroSense digital platform [16]. Through this platform, users can register and associate any of its plots. Various services are then available to support more efficient agricultural production. The platform offers the possibility for the user to perceive, per management zones, specific locations for soil sampling and analysis proposed by the AI algorithm based on satellite or high-resolution drone images. The specified locations are generated according to the assessed variability within the plot and, as such, their number can be lower compared to the number of sampling points obtained from the cell sampling scheme with checkerboard representation [17,18].

In precision agriculture, a management zone defines a sub-region within the same piece of land (plot), which has spatially invariant factors influencing the yield according to which crop management practice is carried out [19,20]. Different types of data have been used for the assessment of spatial variability within the plot such as data about the yield through the years, various soil properties, remotely sensed data, topographic factors, and soil apparent electrical conductivity (ECa) [20,21,22,23]. Among the methods proposed to measure within-plot spatial variability for delineating management zones, a statistical cluster analysis, which integrates various data sources, is the most frequently used as a baseline [24]. The developed AI algorithm focus only on remote sensing data, particularly on multispectral Sentinel-2 satellite images [25,26,27,28].

Management zone estimation within the defined user plot by the AI algorithm is undertaken in two steps: first, by performing the K-means cluster analysis [29,30,31] and then by conducting the spatial filtration with the mode filter on the image created from obtained cluster labels. The K-means takes as input a user-specified parameter K and feature vectors created per minimum area covered by pixels of multispectral images, which is defined by the maximum spatial resolution (10 m × 10 m) achieved by the Sentinel 2 Multispectral Imager (MSI) sensor. The feature vectors can contain various statistics extracted from provided data, such as the vegetation indices [31] calculated from the remote sensing images acquired by satellites or UAVs. For the results presented in this paper, the feature vector contains values of Normalized Difference Vegetation Index (NDVI) calculated using one or more Sentinel-2 images. 

**Figure 2:**
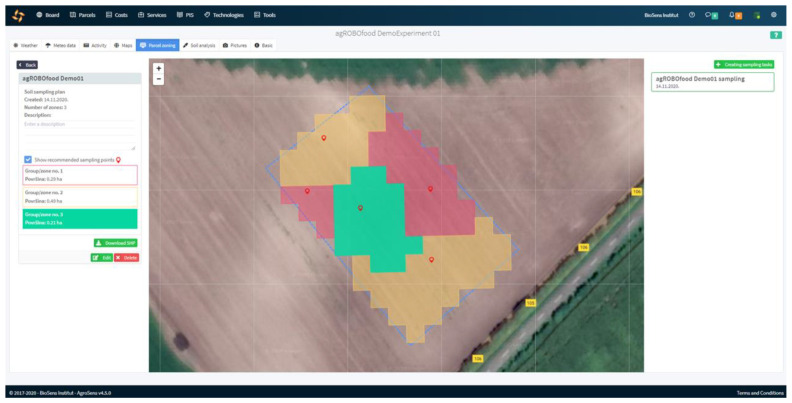
The results of the executed algorithm for proposing optimal sampling points within the AgroSense platform. Each color represents different management zones, while pin markers represent the optimal sampling points per zone.

After generating an image with cluster labels obtained from K-means as pixel values, the second step of the AI algorithm consisting of spatial filtration by taking into account spatial statistics of labels is conducted. This step is necessary to obtain a smooth and more regular shape of boundaries of the management zone and to create connected components within the zones, by correcting the pixel labeling with the inclusion of the spatial context, Figure 2. The final step of the AI algorithm generates sampling points one for each of the connected components within the management zone (e.g., two sampling points for the red management zone since it has two connected components (see Figure 2)). Since the selection of sampling point locations is constrained by the maximal spatial resolution provided by input data, then only the GPS location of the pixel center can be selected as a coordinate for the sampling point. A GPS location of the pixel representing the center of the mass of the associated connected component is selected as the coordinate of the sampling point. The spatial filtration step of the AI algorithm can be repeated several times if the shape of the connected component is such that the center of the mass does not fall within the area of the connected component, Figure 2. The algorithm is implemented in the AgroSense platform as a real-time solution for defining the optimal sampling points of the selected field. The algorithm specifies one sampling point per connected component, but it can generate multiple points per management zone. At this moment, it was decided to optimize the system to cover a larger field area and save the battery for this purpose, and not spend it on additional sampling. The user has the option to manually move, add, and remove suggested sampling points. Once the user finishes the rearrangement of the sampling locations, the soil sampling and analysis task can be saved and downloaded using a smartphone application.

### 2.2. Smartphone Application

**Figure 3:**
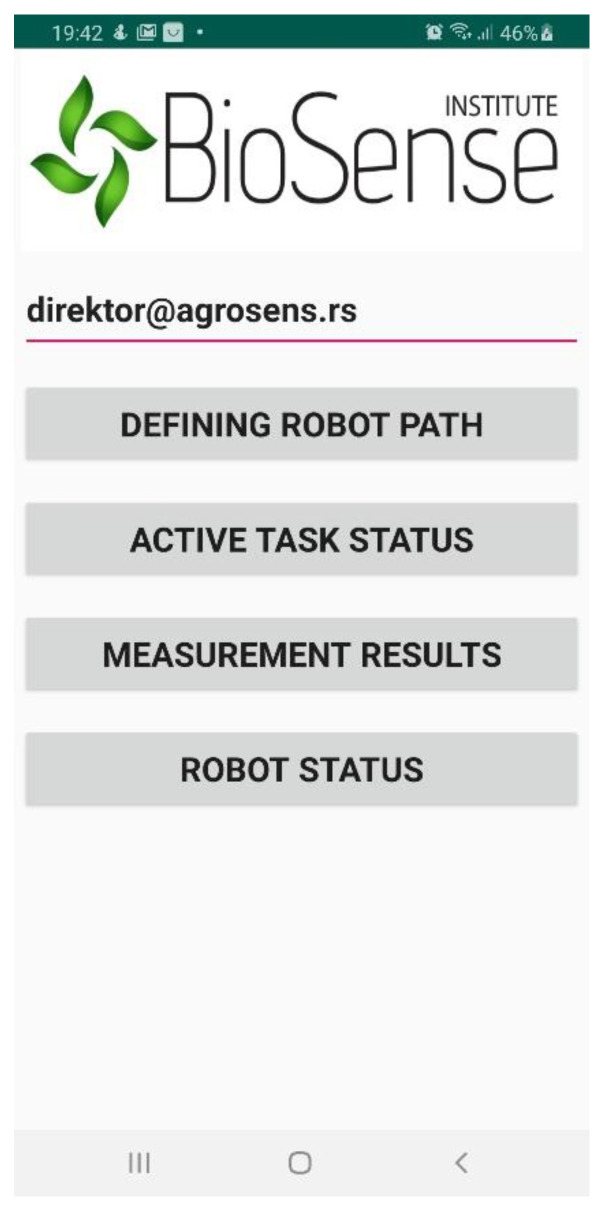
The main menu of the RoboSense smartphone application.

The smartphone application was made for the robot Operator, the well-trained person that monitors the complete soil sampling and analysis procedure performed by a robotic system in the field. The application called RoboSense is designed to be simple to use and to allow the Operator effortless preparation of a new soil sampling task, have an insight into the current state of the active soil sampling task, edit the currently active soil sampling task, if necessary, as well as the possibility of insight into the state of the robotic system, which will be implemented in future work. Figure 3 shows the main menu of the RoboSense smartphone application.

The main menu presents the key functionalities of the application, which are the following:

- A field for entering the email address of the user that defined the soil sampling and analysis task is needed for downloading the task.
- A button “DEFINING ROBOT PATH” is used to define a new robot path.
- A button “ACTIVE TASK STATUS” is used to display the status of the active robot task.
- A button “MEASUREMENT RESULTS” is used to quickly display currently measured nitrate values.
- A button “ROBOT STATUS” is used to display robot status (battery level, water level, and system errors).

### 2.3. Robotic System

The robotic system is a combination of the commercially available UGV platform Husky and a custom-made solution for the task of soil sampling and analysis—minilab. The minilab is mounted on the robotic platform and consists of several main modules:

- Anchoring module.
- Sampling module.
- Sample preparation module.
- Module for soil analysis.

Electronic- and software-wise, a robotic platform is divided into two parts:

- UGV Husky platform, with software based on ROS (Robot Operating System) running on Linux.
- Custom electronics based on ATMega MEGA 2560, with firmware based on C++.

**Figure 4:**
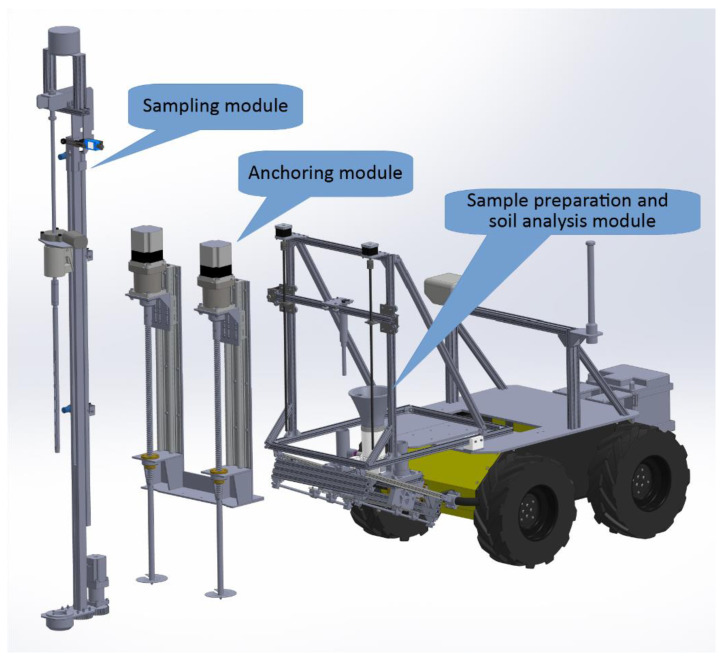
The 3D model of the exploded view of the agrobot Lala model.

The complete model of the robot Lala, with an exploded view of the modules, is shown in Figure 4**.**

The UGV platform’s external dimensions are 990 × 670 × 390 mm. It weighs 50 kg and has a maximum payload of 75 kg. The maximum speed is 1.0 m/s and can achieve typically 3 h of autonomy.

The custom-made solution for soil sampling and analysis fits the UGVs width, and expands 40 cm in front of the UGV, with a total height from the ground of 200 cm. It can take samples from a 30 cm depth and can anchor to 15 cm. It can provide penetration force of up to 720 N. The speed of penetration and extraction, where the most reaction forces are exhibited, is 1 mm/s, while the sampler maximum speed is 3 mm/s. The anchoring speed is 2 mm/s for individual anchors, while the total speed is 1 mm/s concerning their reciprocating motion. While not anchoring, the speed is up to 10 mm/s.

#### 2.3.1. Anchoring Module

**Figure 5:**
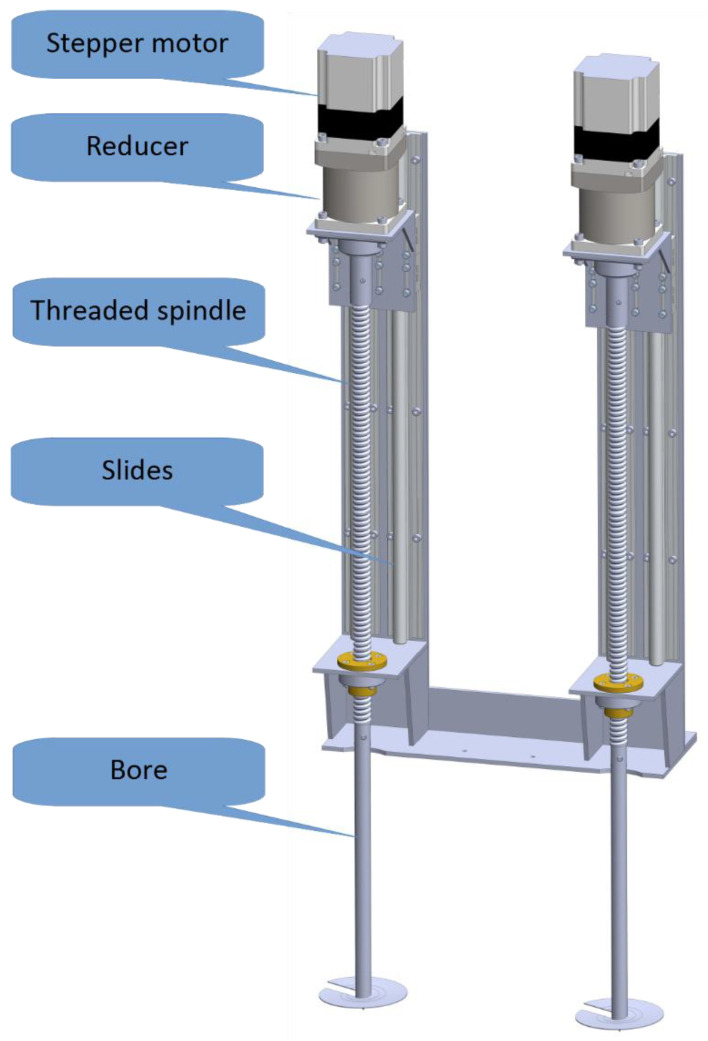
Model of the anchoring module.

During platform development, it was observed that the penetration forces, especially at higher depths, can be large. They can cause an uplift of the whole platform, which leads to an inadequate sample. This means that the volume of the sample is smaller, but the more severe effect is that the sample does not include a part from the required depth. Furthermore, this uplift can cause damage to the equipment, especially the probe, which can be bent or even broken. To prevent this from happening, the platform is equipped with a custom-made anchoring module, which is shown in Figure 5.

The anchoring module consists of two linear stages, driven by the stepper motors (NEMA 34, 6.0 A, 8.7 Nm) over the 6:1 reducer. A threaded spindle with a 20 mm step is connected to the output of the reducer. This large step enables firm anchoring while saving instantaneous power needed for drilling. The threaded spindle moves together with motors on slides. The actuating end of the threaded spindle is extended with the bore, which has a single turn of the thread of approximately 80 mm in diameter, and a step equal to the step of the threaded spindle. This constellation enables smooth and efficient anchoring, without the milling effect on the soil. Again, the bore diameter is optimized between firm anchoring and the instantaneous power needed for the drilling. The estimation of the power needed for anchoring and optimization of the parameters was completed in a manual manner, where a manual thread typically used for round hole excavation is used along with a gauge to measure required forces. Due to the battery power, care is taken to minimize peak and overall power consumption.

The module is equipped with three inductive sensors per drive at dedicated positions. These positions indicate the top position, where the anchors are initialized and transported, a position where drilling starts at ground level, and a position at 15 cm anchoring depth. Besides inductive sensors, the anchoring module is also equipped with two mechanical hardwired end switches per drive for safety reasons at the topmost and bottommost positions.

#### 2.3.2. Sampling Module 

**Figure 6:**
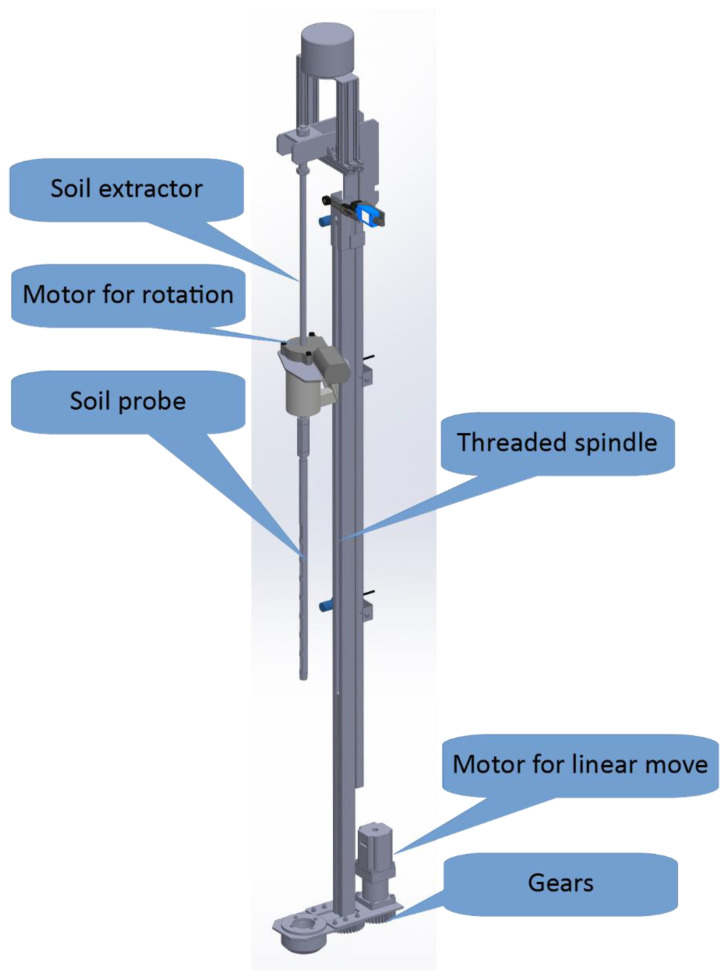
Model of the sampling module.

The sampling module serves to take a soil sample (Figure 6). It is based on the electric probe soil sampling method. Similar to the anchoring module, the sampling module is also a linear stage; however, a threaded spindle and a driving motor are at a fixed location, while the moving part is the soil sampling probe and the probe attachment.

Besides linear movement, the probe also exhibits a rotational move actuated by the motor placed at the top part of the soil probe. This rotational motion is executed before the probe is pulled from the soil at its lowest point. Namely, this move greatly reduces the initial force needed for a sample removal. Moreover, this rotation is implemented during the sample extraction process, where an extractor end is bore designed, therefore, the rotation creates boring, and the soil starts to fall out from the probe openings before it is extracted at the tip of the probe. This significantly reduces the peak force and power needed for the extraction and minimizes the stress on the material of the probe and the extractor. The threaded spindle has a 3 mm step and is driven by a stepper motor NEMA 23, 3 A, 1.8 Nm over a reducer of gear ratio 6:1. The gearbox and the threaded spindle are colinear and connected using two equal gears at the bottom of the module. The module is equipped with four inductive sensors at dedicated positions. These positions indicate the top position, where the extraction is accomplished, a position where the extraction starts, a position where the probe tip is at ground level, and a position at 30 cm sample depth. For safety reasons, the sampling module is equipped with two mechanical hardwired end switches for safety reasons at the topmost and bottommost positions. 

#### 2.3.3. Sample Preparation Module

**Figure 7:**
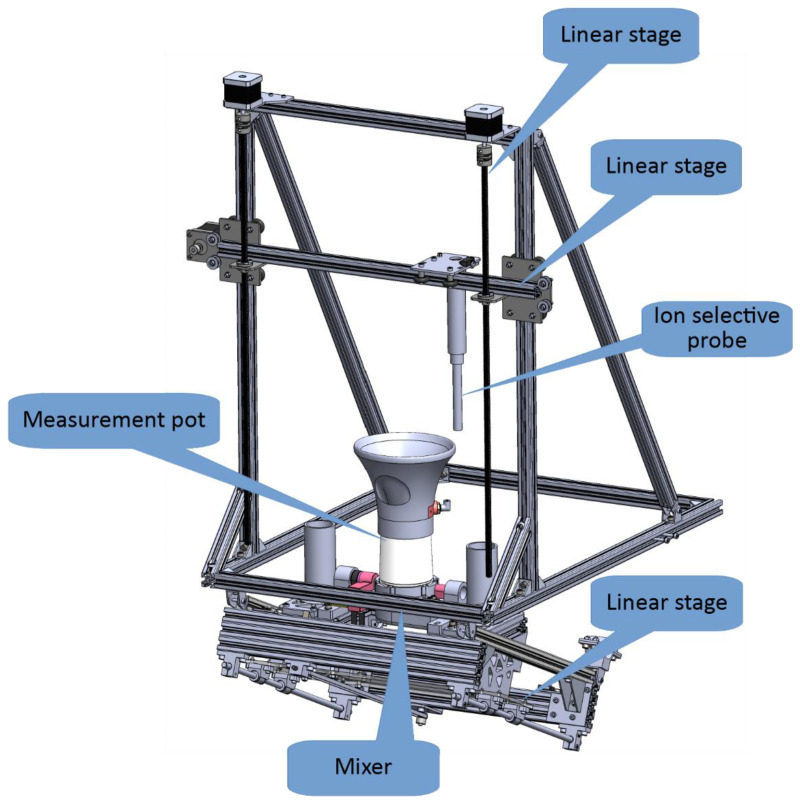
The 3D model of the sample preparation module.

The main purpose of the sample preparation module is to prepare the solution of the soil sample suitable for measurement. It consists of 3-axes linear stages, a measurement pot with one rotational degree of freedom for cleaning purposes, and a mixer. The module is shown in Figure 7.

The ion-selective probe used for soil sample measurement can be maneuvered alongside horizontal and vertical linear stages. Namely, horizontal movement enables the probe to position over the referent solutions or the prepared sample, while during vertical movement the probe can be immersed into referent solutions or the prepared sample. The third linear stage is slightly inclined and is used for platform movement. The moving platform is designed to carry the sample preparation pot and supporting parts. Alongside this stage, the measurement pot can be moved under the probe to collect the extracted soil sample or moved to an initial position where measurements with an ion-selective probe are conducted. During the cleaning process, the pot is moved to the dedicated position, where it can be rotated to pour out the sample solution and be cleaned.

**Figure 8:**
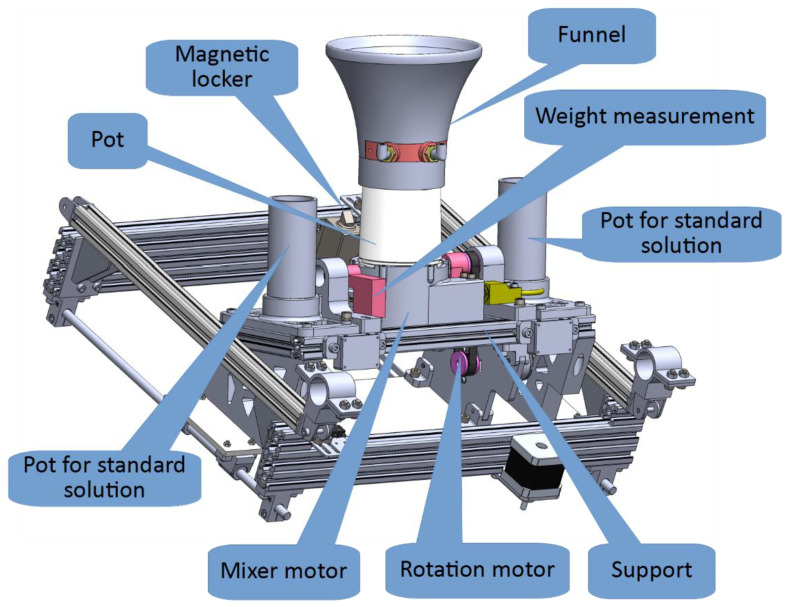
The 3D model of the measurement pot with parts.

The measurement pot consists of the pot, a funnel, support, a weight measurement unit, a motor for rotation, a mixer motor, and a magnetic locker (Figure 8).

The funnel is used to collect falling parts of the soil sample. It also has integrated water channels and nozzles for two purposes. The first one is for the addition of an exact amount of deionized water (DI water), and the other is for cleaning purposes. Those systems are separated and are driven by different pumps. The pump for the precise addition of DI water is less powerful, therefore more easily controllable, while the pump for cleaning is a more powerful pump that can create a shower effect with many nozzles integrated into the funnel. The weight measurement is used to determine the collected soil sample mass and the mass of added DI water, hence their ratio. The amount of added water is predefined to 4:1; however, it can be less if the sample is heavier, and more if the sample is lighter. The magnetic locker secures the platform in the topmost position, enabling the stepper motors to be de-energized to save power. 

Electrically wise, the sample preparation module incorporates:

- Three NEMA 17 stepper motors and associated end switches for linear movement.
- One geared DC motor for the rotation of the pot.
- One brushless motor to mix the sample.
- Two aluminum load cells to measure the soil sample weight, and the weight of added water.
- A magnetic locker.
- Two water pumps with a DI water reservoir.

#### 2.3.4. Module for Soil Analysis

The module for soil analysis is based on an ion-selective electrode (ISE) Vernier NO3-BTA. Besides the electrochemical ISE technique for soil nitrate determination, techniques such as spectrophotometric/spectrometric and biological are available as well. Due to the variable optical signatures of soil, these techniques require considerable site-specific calibration. The biological approach, although promising, should be improved concerning sensor robustness and lifetime. We decided to base our soil analysis module on the ISE probe since it offers the greatest potential for near-term accurate field analysis of nitrates [32].

The operating principle of ISE is based on potentiometry, or more precisely, the measurement of the electric potential difference between referent and indicator electrodes which are immersed in a liquid solution with ions that are measured. The most important part of ion-selective electrodes is an ion-selective membrane that enables the passing of particular ions, in this case, NO3^−^ ions, while preventing the passage of other ones.

The main advantage of ISE nitrates detection is that it does not demand filtration of the sample which significantly simplifies its use in the field.

The module for soil analysis overlaps, to some extent, with the sample preparation module, in the sense that it also uses two linear stages of the sample preparation module to maneuver the probe and the mixer. However, the main part of this module is the ion-selective probe for soil nitrates measurement Vernier NO3-BTA [33]. Before field operation and any reliable and accurate measurement, the probe must be calibrated. For this purpose, a dedicated plane positioning system was developed consisting of the above-mentioned two linear stages, as well as two pots for standard solutions installed in the module. The calibration procedure is completed automatically on the robotic platform by using 50 and 200 mg/L calibration standards before each soil sampling and analysis task. Based on measurements of standard solutions, a calibration curve of exponential nature is derived. Subsequent measurements of the nitrates are then derived by inserting measured ADC values in this equation. The exact concentration of nitrates is also determined by the proportion of the soil sample mass and the mass of the added water, used to make an appropriate solution of the sample. This ratio is 4:1 in favor of water. Currently, a measurement of soil moisture is not implemented, and this can cause a small error in the measurement.

During the measurement, the sample solution is constantly mixed with a lower mixing speed to achieve a homogeneous solution. The measurement process takes 3.5 min, which is the time needed for the probe to stabilize the response.

**Figure 9:**
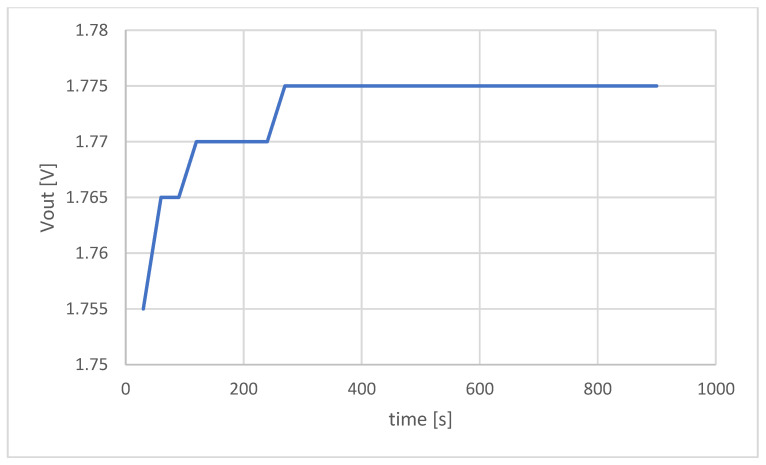
Dynamic response of the ISE probe for the nitrate standard concentration of 200 mg/L.

Before integrating the module for soil analysis with the robotic platform, a series of benchtop laboratory tests were performed to optimize measurements and to test the accuracy of the proposed method against the referent one. To optimize the measurement time, the ISE probe was immersed in a 200 mg/L nitrate solution to determine dynamic output. As can be seen in Figure 9, the output voltage stabilized after 210 s; therefore, this measurement time was used in the continuation of the measurement.

**Figure 10:**
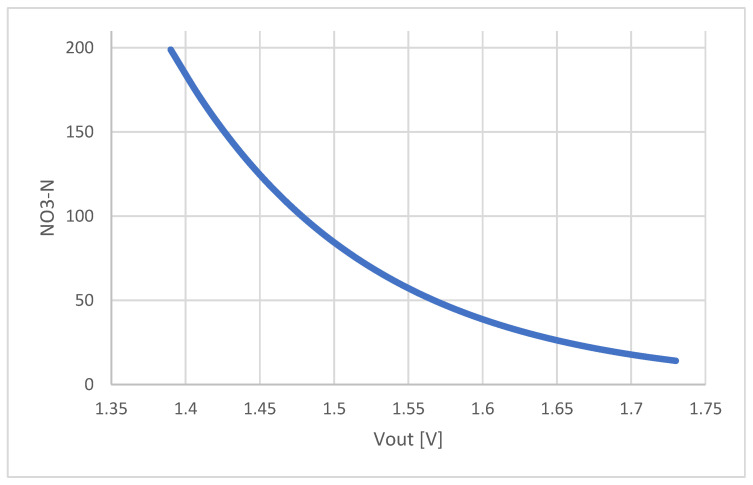
Calibration curve that relates ISE probe voltage output with nitrate-nitrogen concentration.

Afterward, a series of nitrate standards were prepared to calibrate the ISE probe. The nitrate concentrations were chosen to cover expected nitrate values in the field and were the following: 0, 6.25, 12.5, 25, 50, 100, 150, and 200 mg/L. The measurements were performed with each standard and the calibration curve was constructed (Figure 10).

The relation between nitrate concentration and ISE probe voltage output is exponential:

C_N_ = a × e^bV^_S_, (1)

where C_N_ is nitrate concentration [mg/L], V_S_ is the voltage output of ISE probe [V], while a and b are coefficients determined by calibration. From the nature of this relation, it is clear that only two nitrate standards are sufficient to determine the calibration curve. This is also beneficial for the in-field system since there is no need to have all the calibration standards on the platform for the on-site calibration. By analyzing the errors between the calibration curve constructed with all the stated standards and the ones constructed with the help of all the possible standard pairs, it was concluded that the combination of 50 and 200 mg/L gives the lowest relative error with the respect to the full range which was 2.95%.

To test the calibration curve, 15 agricultural soil samples were used, and the results of nitrates measurements were cross compared to the results obtained according to the Bremner method for determination of inorganic nitrogen [34]. The comparison of results is presented in Table 1.

**Figure 11:**
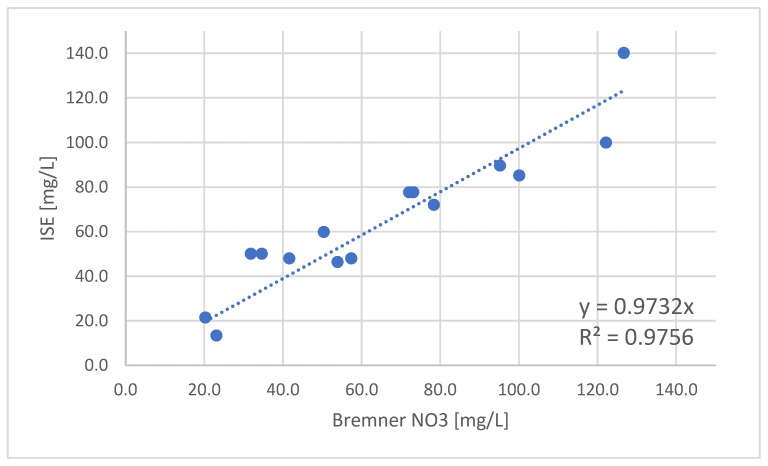
Comparison between the referent Bremner method and ISE method for nitrate-nitrogen detection.

The results are compared in graphical form, where the regression line that describes the actual relationship between the two methods is presented (Figure 11). It can be seen that the regression slope is 0.97, while the R^2^ factor is 0.97 based on which we can confirm that this method of calibration is validated.

#### 2.3.5. ROS Implementation

The main control of the platform is implemented through Robotic Operating System (ROS) running under the Linux platform. It comes with preinstalled drivers for all sensors provided by the vendor: LIDAR, Inertial Measurement Unit (IMU), motor drivers with sensors, and RTK GPS module. A ROS navigation package from the manufacturer is preinstalled and used for platform navigation in the field. A module that controls the whole system is developed in-house. This module receives and executes the task from the cloud server. It constantly updates the status of the task and each individual sample point within the task. This status can be monitored by a user (robot Operator) over a dedicated application on a smartphone.

Four ROS nodes are created to communicate with the custom-made electronics for the minilab. The first one publishes control messages for the minilab. The second one receives the responses to the issued commands published by the minilab. The third one receives the measurement results of the nitrate concentration. The last one is used to publish debugging information for the user to monitor the task execution.

There is a substantial list of ROS packages that are installed and used in the robotic platform; therefore, we just want to mention the most important ones in the following list sorted in alphabetical order:

- actionlib—provides a standardized interface for interfacing with preemptable tasks.
- amcl—a probabilistic localization system for a robot moving in 2D.
- costmap 2D—provides an implementation of a 2D costmap that takes in sensor data from the world, builds a 2D or 3D occupancy grid of the data.
- imu_tools—contains IMU-related filters and visualizers.
- navigation—a 2D navigation stack that takes in information from odometry, sensor streams, and a goal pose and outputs safe velocity commands that are sent to a mobile base.
- move_base—provides an implementation of an action that, given a goal in the world, will attempt to reach it with a mobile base.
- nmea_comms and nmea_msgs—for interfacing GPS.
- rosserial—for wrapping standard ROS serialized messages and multiplexing multiple topics and services over a character device such as a serial port or network socket.
- tf2—lets the user keep track of multiple coordinate frames over time.

#### 2.3.6. Electronics

Hardware-wise, the electronics is based on Arduino ATMega MEGA 2560, and a firmware based on C++. The firmware controls all modules of the minilab, including their actuators and sensors. It has two serial interfaces to the external world. The first one is with the ROS platform. This is the main interface during the operation. The second interface serves for debugging and manual control purposes. It can be directly wire-interfaced, or more suitably over a Bluetooth (BT) wireless module, where the status and debug messages can be monitored during the whole process on a smartphone application. This interface, besides high-level commands for the whole processes, also allows low-level commands. The low-level commands are used for testing the individual features, but also for the manual control of the minilab in case of errors during normal operation.

#### 2.3.7. An Overview of the System

**Figure 12:**
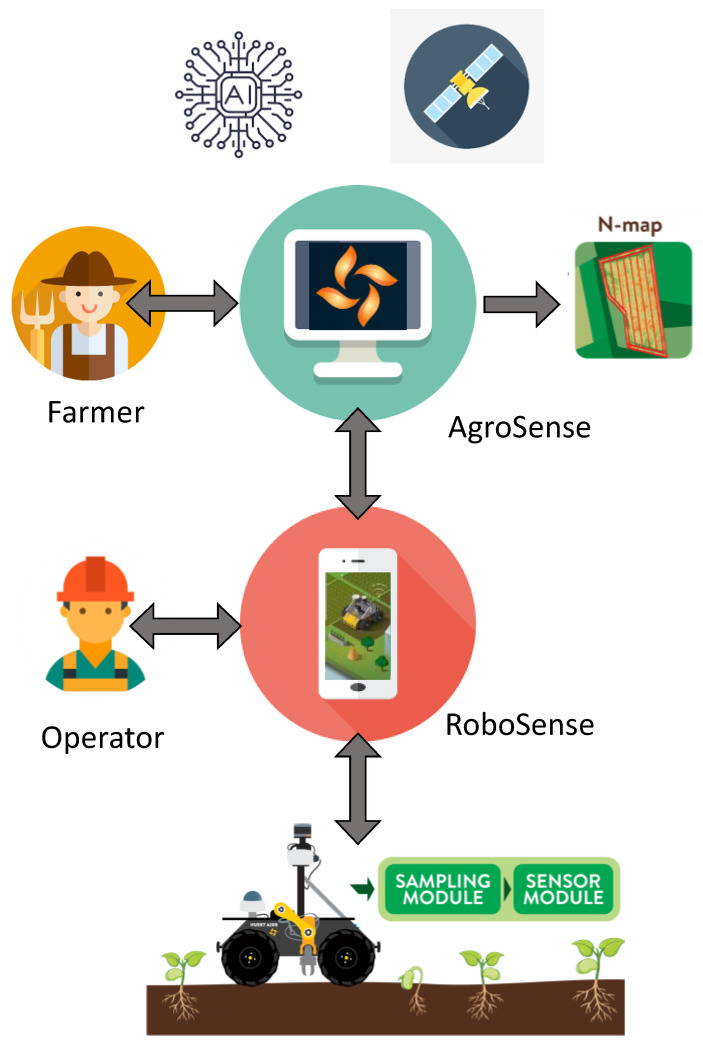
An overview of the system for soil sampling and analysis.

An overview of the system is presented in Figure 12, based on which the basic procedure can be summarized in the following steps:

1. A farmer defines soil sampling and analysis tasks with the help of the AgroSense platform.
2. The Operator downloads the specific task from the AgroSense platform with the help of the RoboSense smartphone application and defines the optimal route.
3. The task prepared by the Operator is uploaded to the robotic system via the RoboSense server.
4. The robotic system performs the soil sampling and analysis task and during the process, the status of robotic system operations is being refreshed.
5. Once the task is finished, the Operator uploads the measurement results to the AgroSense platform.
6. A farmer selects the task to visualize the results for nitrogen content measurements.
7. A farmer creates a fertilization prescription map for the desired type of fertilizer.

The cost of the entire robotic system is around EUR 50,000. The major expense is a commercial UGV platform, which costs about EUR 40,000. The rest goes to custom-made equipment. With this high cost, a targeted business model for this platform is to be provided as a service, especially having in mind the integration with the AgroSense platform which also provides agricultural services.

## 3. Results

The complete system was tested on the plot within the field of the commercial farm in the locality Krivaja, Serbia. The size of the plot was 1 ha. Plowing and tilling the plot were completed before performing the test of the robotic system.

The process started by defining the soil sampling and analysis task with the help of the cloud-based platform AgroSense. After defining the experimental plot and choosing the relevant satellite image, the algorithm for proposing the locations for soil sampling was initiated. As a result, in total, five points determining sampling locations were estimated within three different management zones (Figure 2). In this way, the soil sampling and analysis task was created, as can be seen in the upper right corner of Figure 2.

**Figure 13:**
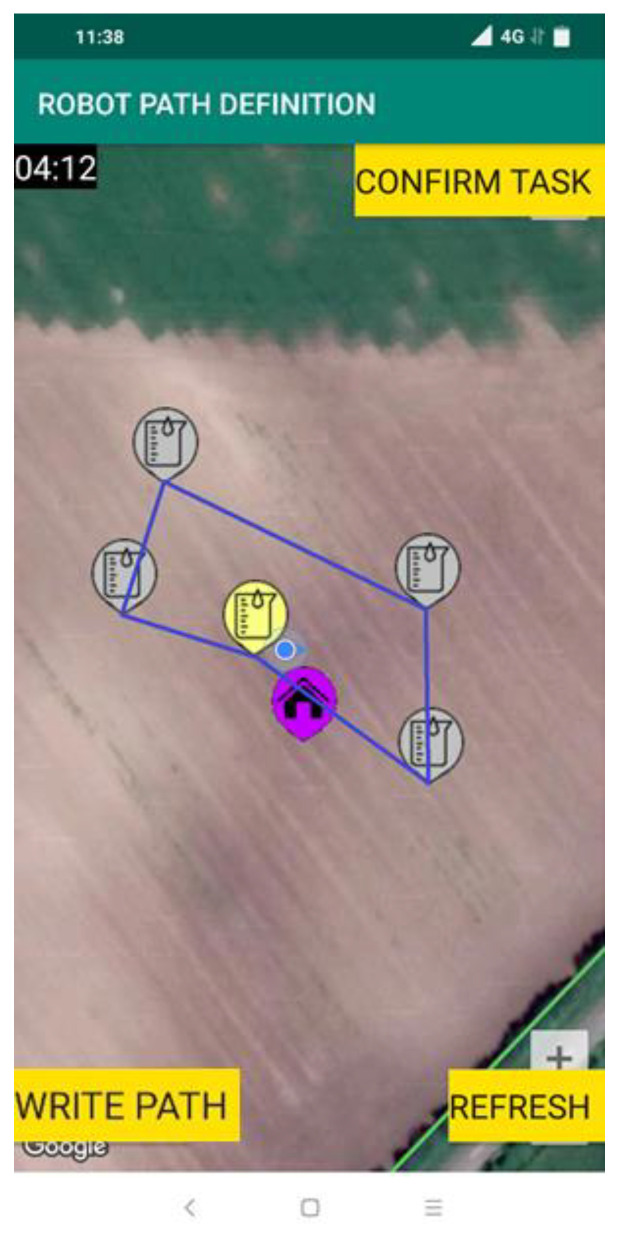
Google map with sampling points and optimal route.

The task created in this way was downloaded using the smartphone application to initiate the soil sampling and analysis process. When the soil sampling task was opened, a Google map was displayed showing the defined sampling points in the form of markers. Next, the optimal route for the robotic system was created as shown by blue connecting lines in Figure 13.

**Figure 14:**
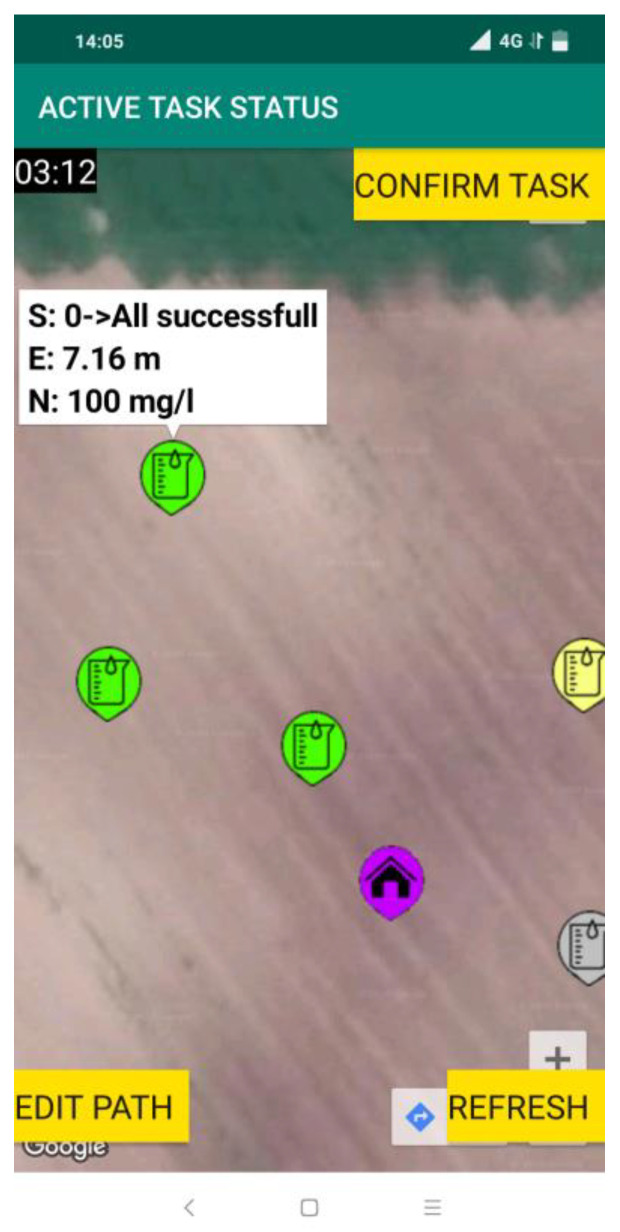
Active task status.

It is important to note that if there is a need, the route and sampling points can be edited by the Operator, which was not done in this example case. In this case, the route was confirmed and uploaded to the cloud-based platform by pressing the “WRITE PATH” button. Next, the robotic system was initiated by the Operator, meaning that the system entered the procedure for soil sampling and analysis. First, the robotic system completed the initialization of each module, afterward, the soil sampling and analysis task was downloaded from the AgroSense server. Next, the robotic system visited the first point in the downloaded list. Before the sampling was completed the robotic system performed the calibration of the ion-selective electrode for measuring nitrate content. After the calibration process, the robotic system undertook routines of anchoring, soil sampling, sample preparation, analysis of the sample, cleaning, un-anchoring, and moving to the next location. The movement of the robotic system was stable, and it successfully overcame all the bumps in the field. If the plot was only plowed, furrow would cause stability issues. The robotic system regularly updated the status of each step of the soil sampling and analysis procedure by sending messages to the AgroSense server, which could be seen by choosing the “ACTIVE TASK STATUS” option from the main menu. Depending on the status, the sampling points were displayed with markers of different colors (Figure 14). The sampling points that have the initial status are shown in gray, meaning the robot has not yet visited the point. The yellow color represents the sampling point at which the robot was currently engaged. The sampling points at which all sampling tasks were performed successfully were shown in green. By clicking on the sampling point marker for a given point additional information such as the current status of the sampling point (status code and brief status description), robot positioning error in meters (distance between specified and achieved robot coordinates), and measured nitrate value if available was displayed.

When the robot successfully visited all sampling points by clicking on the “CONFIRM TASK” button the Operator confirms that the active task on the parcel has been completed and the measurement results become available on the AgroSense platform.

The measurement results of this example case are presented in Table 2.

**Figure 15:**
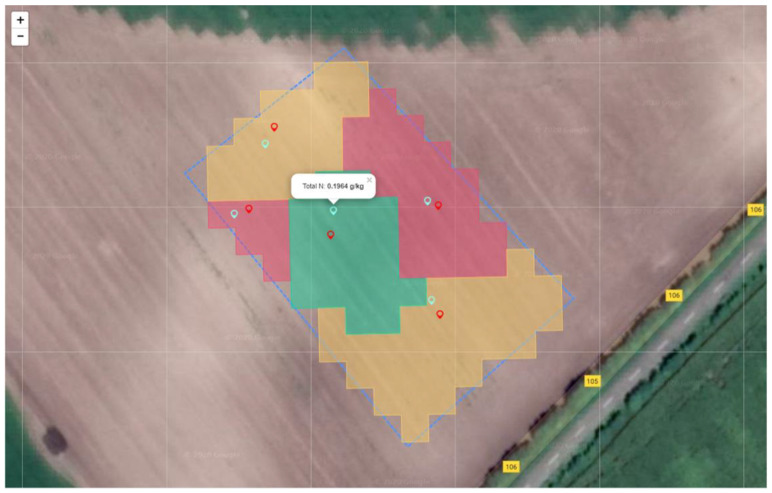
Fertilization prescription map generated based on the measurement results. Red pins represent proposed sampling locations by AI algorithm, while green pins represent actual sampling locations.

Based on the measurement results, a fertilization prescription map was generated (Figure 15). Fertilization prescription was completed based on the optimal value of nitrogen in the field which was taken to be 30 ppm. This was just above the critical value of nitrate-nitrogen needed for the maize [35]. Based on the in-field measurement results and nitrogen target value, the amount of missing nitrogen was determined and prescribed to be applied. Green pins represent the locations of the actual sampling points which are different from the proposed ones due to the positioning error.

## 4. Discussion

We presented a complete system for soil sampling and analysis intended to help farmers to optimize fertilization in their fields and, in this way, be more efficient in a sustainable way. An overview of the system is presented in Figure 12. The presentation of the system and its functionalities can be seen in a promotional video [36]. The system consists of a user-oriented platform called AgroSense which the farmer uses to define the soil sampling and analysis task with the help of high-resolution satellite images and an AI algorithm. The same platform is used for the visualization of the measurement results and obtaining the fertilization prescription. The next component of the system is the RoboSense application which is used by the Operator in the field to monitor the soil sampling and analysis task that is performed by the robotic system.

The system was tested in an operational environment in the 1 ha experimental field, during which all the stated functionalities of the system were successfully demonstrated. The complete task took 165 min to be completed, during which five samples were taken. The measurement results showed slightly higher values of nitrate-nitrogen than usual [37] which can be explained by the fact that two days before the tests, the base fertilization was performed.

It should be noted that the fertilization prescription algorithm was tested previously, on another 5 ha experimental field. In this case, soil samples were taken from the field and tested for nitrate concentration with the same ISE probe. Based on the results, a fertilization prescription was completed. The average yield of the experimental field was 1.76% higher in comparison to the entire plot, which is a promising result but should be repeated in future seasons. It should be noted that at the same time more than 7.5% of KAN fertilizer was saved.

At this level, all the functionalities of the system were successfully demonstrated in an operational environment, but it still needs to be further tested to include different real-life scenarios and environmental conditions before it could become commercially available. Furthermore, it should be noted that soil sampling analysis was not corrected for the soil moisture content but mixing the soil sample with water by a ratio of 1:4 reduces the soil moisture influence. In the future, a soil moisture sensor could be integrated into the platform to have more accurate measurements. In addition, since the main challenge of taking the sample from the soil is resolved, a different sensor such as multi-ion probes could be used on the same sample to measure the rest of the important soil nutrients by detecting ions such as Ca^2+^, K^+^, Mg^2+^, NH^4+^, NO^3−^, P[HPO_4_^2−^], Cl^−^, and Na^+^ [38], together with parameters such as pH and electrical conductivity.

Since the robotic system is equipped with RTK GPS, it could be used for ground-based topographic mapping to generate high-resolution elevation data at the landscape level. The generated maps are highly useful in the understanding of soil water and nutrient movement and would represent an additional valuable level of information for farmers.

In the future, the autonomy of the robotic system could be improved by optimizing soil sampling and analysis procedures as well as by using an advanced battery solution that is based on LiFePo4 technology which can offer up to 10 times more capacity.

## 5. Conclusions

This paper represents Agrobot Lala—an autonomous robotic system for real-time, in-field soil sampling and analysis of nitrates. The presented solution helps with fertilization optimization which leads to more efficient production in a sustainable way. The system comprises a cloud-based application for task management and generation of sample points on the plot based on proprietary algorithms of artificial intelligence (AI), a smartphone application for monitoring and customization of the tasks, and a robotic system for fieldwork of acquiring and analysis of soil samples.

With the help of the system, a farmer defines soil sampling and analysis tasks with the help of a cloud-based platform and AI algorithm. The task is performed by the robotic system providing the measurement results in real-time. As the result of the analysis, a fertilization prescription is generated which reached more than 7.5% of KAN fertilizer savings and showed a 1.76% yield improvement during the initial test. All the stated functionalities of the system were verified on the 1 ha experimental field by taking and analyzing 5 soil samples.

The presented system can be significantly improved by introducing a multi-ion probe for detecting additional important nutrients in the soil besides nitrates, as well as additional sensors for pH and electrical conductivity measurements.

## Figures and Tables

**Table 1:** Comparison between ISE probe nitrate-nitrogen measurement results and results obtained according to the Bremner method.

Sample	Bremner NO3-N [mg/L]	ISE NO3-N [mg/L]
1	20.3	21.4
2	23.1	13.3
3	31.9	50
4	34.7	50
5	41.7	47.9
6	50.4	59.8
7	53.9	46.3
8	57.4	47.9
9	72.1	77.6
10	73.2	77.6
11	78.4	71.9
12	95.2	89.5
13	100.1	85.1
14	122.2	99.9
15	126.7	140.1

**Table 2:** The measurement results from the test performed on the plot within the field of the commercial farm in the locality Krivaja, Serbia.

Sample	Mass [g]	NO3 [mg/L]	NO3-N [kg N ha^−1^]
1	37.06	196.38	177.00
2	23.27	175.01	157.74
3	36.05	85.07	76.68
4	45.52	91.70	82.65
5	33.35	98.64	88.91

## Data Availability

Data is contained within the article.
